# (2*E*)-3-(3,4-Dimeth­oxy­phen­yl)-1-(2,5-dimethyl­thio­phen-3-yl)prop-2-en-1-one

**DOI:** 10.1107/S1600536810029272

**Published:** 2010-07-31

**Authors:** Abdullah M. Asiri, Salman A. Khan, M. Nawaz Tahir

**Affiliations:** a The Center of Excellence for Advanced Materials Research, King Abdul Aziz University, Jeddah 21589, PO Box 80203, Saudi Arabia; b Department of Chemistry, Faculty of Science, King Abdul Aziz University, Jeddah 21589, PO Box 80203, Saudi Arabia; c Department of Physics, University of Sargodha, Sargodha, Pakistan

## Abstract

The mol­ecule of the title compound, C_17_H_18_O_3_S, is essentially planar: the phenyl and thio­phene rings form a dihedral angle of 2.79 (10)° and they are inclined to the central propenone unit by 6.20 (15) and 4.78 (15)°, respectively. In the crystal, mol­ecules are connected into dimers *via* pairs of C—H⋯O inter­actions, generating *R*_2_^2^(14) motifs. π–π stacking inter­actions between the thio­phene rings also occur, with a centroid–centroid distance of 3.8062 (12) Å.

## Related literature

For background to chalcones, their activity and applications, see: Bandgar *et al.* (2010 ▶); Deng *et al.* (2007 ▶); Liu *et al.* (2003 ▶); Verma *et al.* (2007 ▶). For graph-set notation, see: Bernstein *et al.* (1995 ▶).
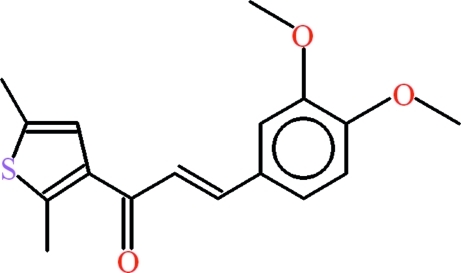


## Experimental

### 

#### Crystal data

C_17_H_18_O_3_S
*M* *_r_* = 302.37
Monoclinic, 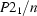

*a* = 9.1821 (6) Å
*b* = 8.3529 (5) Å
*c* = 20.3443 (13) Å
β = 94.624 (4)°
*V* = 1555.27 (17) Å^3^
*Z* = 4
Mo *K*α radiation
μ = 0.22 mm^−1^
*T* = 296 K
0.30 × 0.24 × 0.22 mm

#### Data collection

Bruker Kappa APEXII CCD diffractometer
Absorption correction: multi-scan (*SADABS*; Bruker, 2005 ▶) *T*_min_ = 0.868, *T*_max_ = 0.965
11371 measured reflections
2791 independent reflections
2182 reflections with *I* > 2σ(*I*)
*R*_int_ = 0.025

#### Refinement

*R*[*F* ^2^ > 2σ(*F* ^2^)] = 0.036
*wR*(*F* ^2^) = 0.106
*S* = 1.07
2791 reflections
191 parameters
H-atom parameters constrained
Δρ_max_ = 0.15 e Å^−3^
Δρ_min_ = −0.24 e Å^−3^

### 

Data collection: *APEX2* (Bruker, 2009 ▶); cell refinement: *SAINT* (Bruker, 2009 ▶); data reduction: *SAINT*; program(s) used to solve structure: *SHELXS97* (Sheldrick, 2008 ▶); program(s) used to refine structure: *SHELXL97* (Sheldrick, 2008 ▶); molecular graphics: *ORTEP-3 for Windows* (Farrugia, 1997 ▶) and *PLATON* (Spek, 2009 ▶); software used to prepare material for publication: *WinGX* (Farrugia, 1999 ▶) and *PLATON*.

## Supplementary Material

Crystal structure: contains datablocks text, I. DOI: 10.1107/S1600536810029272/gk2297sup1.cif

Structure factors: contains datablocks I. DOI: 10.1107/S1600536810029272/gk2297Isup2.hkl

Additional supplementary materials:  crystallographic information; 3D view; checkCIF report

## Figures and Tables

**Table 1:** Hydrogen-bond geometry (Å, °)

*D*—H⋯*A*	*D*—H	H⋯*A*	*D*⋯*A*	*D*—H⋯*A*
C6—H6⋯O3^i^	0.93	2.41	3.175 (2)	139

Symmetry code: (i) 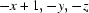.

**Fig. 1.**
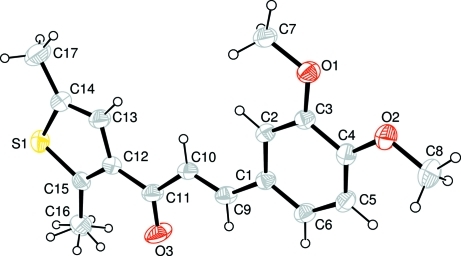
View of the title compound with the atom numbering scheme. The displacement ellipsoids are drawn at the 30% probability level. H-atoms are shown as small spheres of arbitrary radii.

**Fig. 2.**
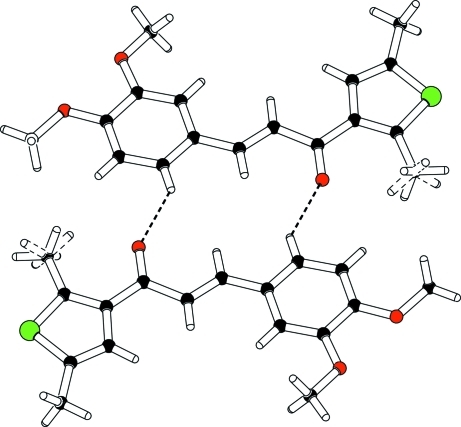
The dimers with R22(14) ring motif.

## supplementary crystallographic information

### Comment

α,β- Unsaturated ketones are a family of bicyclic flavonoids, defined by the presence of two benzene rings joined by a three carbon bridge. Many natural or synthetic chalcones, as well as chalcone glucosides and dimeric chalcones, were found to show diverse pharmacological effects, such as antimicrobial activity (Bandgar *et al.*, 2010), anti-HIV-1 protease activity (Deng *et al.*, 2007) and antileishmanial activity (Liu *et al.*, 2003). In addition, chalcones were used as important intermediates for the total synthesis of some natural products (Verma *et al.*, 2007). On the bases of these aspects, we herein report the synthesis and crystal structure of title compound (Fig. 1).

In the title compound, the group A (C1—C6/O1/O2) of 3,4-dimethoxyphenyl, the central group B (C9—C11/O3) and group C (C12—C17/S1) of 2,5-dimethylthiophen-3-yl moiety are planar. The dihedral angle between A/B, A/C and B/C is 6.58 (14), 3.19 (8) and 4.78 (15)°, respectively. The C-atoms, C7 and C8 of methoxy groups are at a distance of -0.1564 (27) and -0.0979 (32) Å from the mean square plane of the group A. The title compound consists of dimers which are formed due to C—H···O type of intermolecular H-bonding (Table 1, Fig. 2) and complete *R*_2_^2^(14) ring motif (Bernstein *et al.*, 1995). The π···π stacking interactions between their thiophene rings is also present, with the centroid-to centroid distance of 3.8062 (12) Å [symmetry code: - *x*, 1 - *y*, - *z*].

### Experimental

A solution of 3-acetyl-2,5-dimethythiophene (0.38 g, 2.5 mmol) and 3,4-dimethoxybenzaldehyde (0.41 g, 2.5 mmol) in ethanolic solution of NaOH (3.0 g in 10 ml of methanol) was stirred for 16 h at room temperature. The solution was poured into ice cold water of pH = 2 (pH adjusted by HCl). The solid was separated and dissolved in CH_2_Cl_2_, washed with saturated solution of NaHCO_3_ and evaporated to dryness. The residual was recrystallized from methanol/chloroform to affoard light yellow prisms . Yield: 76%; m.p. 387–388 K. IR (KBr) \v_max_ cm^-1^: 2909 (C—H), 1647 (C═O), 1583(C═C).

### Refinement

The H-atoms were positioned geometrically (C–H = 0.93–0.96 Å) and refined as riding with *U*_iso_(H) = x*U*_eq_(C), where x = 1.5 for methyl and x = 1.2 for aryl H-atoms. One of the methyl group is disordered over two positions related by a rotation of 60° around the C-C bond.

### Crystal data

**Table:**

C_17_H_18_O_3_S	*F*(000) = 640
*M**_r_* = 302.37	*D*_x_ = 1.291 Mg m^−^^3^
Monoclinic, *P*2_1_/*n*	Mo *K*α radiation, λ = 0.71073 Å
Hall symbol: -P 2yn	Cell parameters from 2182 reflections
*a* = 9.1821 (6) Å	θ = 2.5–25.3°
*b* = 8.3529 (5) Å	µ = 0.22 mm^−^^1^
*c* = 20.3443 (13) Å	*T* = 296 K
β = 94.624 (4)°	Prism, yellow
*V* = 1555.27 (17) Å^3^	0.30 × 0.24 × 0.22 mm
*Z* = 4	

### Data collection

**Table:**

Bruker KAPPA APEXII CCD diffractometer	2791 independent reflections
Radiation source: fine-focus sealed tube	2182 reflections with *I* > 2σ(*I*)
graphite	*R*_int_ = 0.025
Detector resolution: 8.10 pixels mm^-1^	θ_max_ = 25.3°, θ_min_ = 2.5°
ω scans	*h* = −10→11
Absorption correction: multi-scan (*SADABS*; Bruker, 2005)	*k* = −9→10
*T*_min_ = 0.868, *T*_max_ = 0.965	*l* = −24→24
11371 measured reflections	

### Refinement

**Table:**

Refinement on *F*^2^	Primary atom site location: structure-invariant direct methods
Least-squares matrix: full	Secondary atom site location: difference Fourier map
*R*[*F*^2^ > 2σ(*F*^2^)] = 0.036	Hydrogen site location: inferred from neighbouring sites
*wR*(*F*^2^) = 0.106	H-atom parameters constrained
*S* = 1.07	*w* = 1/[σ^2^(*F*_o_^2^) + (0.0462*P*)^2^ + 0.374*P*] where *P* = (*F*_o_^2^ + 2*F*_c_^2^)/3
2791 reflections	(Δ/σ)_max_ < 0.001
191 parameters	Δρ_max_ = 0.15 e Å^−^^3^
0 restraints	Δρ_min_ = −0.24 e Å^−^^3^

### Special details

**Table:**

Geometry. Bond distances, angles *etc*. have been calculated using the rounded fractional coordinates. All su's are estimated from the variances of the (full) variance-covariance matrix. The cell e.s.d.'s are taken into account in the estimation of distances, angles and torsion angles
Refinement. Refinement of *F*^2^ against ALL reflections. The weighted *R*-factor *wR* and goodness of fit *S* are based on *F*^2^, conventional *R*-factors *R* are based on *F*, with *F* set to zero for negative *F*^2^. The threshold expression of *F*^2^ > σ(*F*^2^) is used only for calculating *R*-factors(gt) *etc*. and is not relevant to the choice of reflections for refinement. *R*-factors based on *F*^2^ are statistically about twice as large as those based on *F*, and *R*- factors based on ALL data will be even larger.

### Fractional atomic coordinates and isotropic or equivalent isotropic displacement parameters (Å^2^)

**Table:**

	*x*	*y*	*z*	*U*_iso_*/*U*_eq_	Occ. (<1)
S1	−0.23565 (6)	0.41751 (7)	−0.05399 (3)	0.0660 (2)	
O1	0.29637 (14)	−0.19408 (15)	0.29377 (6)	0.0576 (4)	
O2	0.54659 (15)	−0.32480 (16)	0.28650 (7)	0.0659 (5)	
O3	0.20712 (19)	0.1767 (2)	−0.04839 (7)	0.0981 (7)	
C1	0.37251 (18)	−0.05442 (19)	0.12728 (8)	0.0467 (6)	
C2	0.29878 (18)	−0.07466 (19)	0.18470 (8)	0.0450 (5)	
C3	0.36019 (19)	−0.16436 (19)	0.23649 (8)	0.0451 (5)	
C4	0.49784 (19)	−0.2356 (2)	0.23254 (9)	0.0490 (6)	
C5	0.57073 (19)	−0.2145 (2)	0.17692 (10)	0.0551 (6)	
C6	0.50821 (19)	−0.1245 (2)	0.12462 (9)	0.0541 (6)	
C7	0.1508 (2)	−0.1415 (3)	0.29816 (9)	0.0614 (7)	
C8	0.6814 (3)	−0.4081 (3)	0.28383 (12)	0.0842 (9)	
C9	0.3079 (2)	0.0318 (2)	0.06976 (9)	0.0543 (6)	
C10	0.1823 (2)	0.1089 (2)	0.06199 (8)	0.0518 (6)	
C11	0.1314 (2)	0.1829 (2)	−0.00184 (9)	0.0582 (7)	
C12	−0.0124 (2)	0.2618 (2)	−0.00808 (8)	0.0512 (6)	
C13	−0.1133 (2)	0.2654 (3)	0.04167 (9)	0.0626 (7)	
C14	−0.2388 (2)	0.3431 (3)	0.02449 (10)	0.0645 (7)	
C15	−0.0661 (2)	0.3419 (2)	−0.06373 (9)	0.0534 (6)	
C16	0.00200 (19)	0.3678 (3)	−0.12765 (8)	0.0764 (9)	
C17	−0.3685 (2)	0.3698 (3)	0.06374 (9)	0.0992 (11)	
H2	0.20796	−0.02712	0.18767	0.0540*	
H5	0.66227	−0.26053	0.17423	0.0661*	
H6	0.55856	−0.11126	0.08712	0.0649*	
H7A	0.08903	−0.18692	0.26264	0.0921*	
H7B	0.14723	−0.02682	0.29529	0.0921*	
H7C	0.11750	−0.17510	0.33951	0.0921*	
H8A	0.75919	−0.33235	0.28076	0.1263*	
H8B	0.67593	−0.47691	0.24591	0.1263*	
H8C	0.69998	−0.47129	0.32304	0.1263*	
H9	0.36315	0.03252	0.03340	0.0652*	
H10	0.12456	0.11680	0.09736	0.0621*	
H13	−0.09372	0.21771	0.08274	0.0751*	
H16A	0.01848	0.26624	−0.14783	0.1146*	0.800
H16B	0.09342	0.42280	−0.11923	0.1146*	0.800
H16C	−0.06243	0.43103	−0.15670	0.1146*	0.800
H17A	−0.35355	0.31512	0.10525	0.1488*	
H17B	−0.45484	0.32891	0.03968	0.1488*	
H17C	−0.38004	0.48231	0.07136	0.1488*	
H16D	−0.06913	0.34716	−0.16386	0.1146*	0.200
H16E	0.08332	0.29641	−0.13003	0.1146*	0.200
H16F	0.03529	0.47650	−0.12987	0.1146*	0.200

### Atomic displacement parameters (Å^2^)

**Table:**

	*U*^11^	*U*^22^	*U*^33^	*U*^12^	*U*^13^	*U*^23^
S1	0.0546 (3)	0.0741 (4)	0.0675 (3)	0.0037 (3)	−0.0054 (2)	−0.0005 (3)
O1	0.0578 (8)	0.0640 (8)	0.0522 (7)	0.0112 (6)	0.0117 (6)	0.0113 (6)
O2	0.0603 (8)	0.0686 (9)	0.0679 (9)	0.0191 (7)	−0.0009 (7)	0.0068 (7)
O3	0.0874 (11)	0.1509 (16)	0.0607 (9)	0.0547 (11)	0.0350 (8)	0.0337 (10)
C1	0.0462 (10)	0.0443 (9)	0.0504 (10)	−0.0022 (7)	0.0085 (8)	−0.0028 (7)
C2	0.0419 (9)	0.0426 (9)	0.0512 (10)	0.0027 (7)	0.0080 (7)	−0.0023 (7)
C3	0.0468 (10)	0.0402 (9)	0.0489 (9)	−0.0008 (7)	0.0068 (8)	−0.0029 (7)
C4	0.0462 (10)	0.0434 (9)	0.0565 (10)	0.0007 (8)	−0.0014 (8)	−0.0028 (8)
C5	0.0390 (10)	0.0560 (11)	0.0706 (12)	0.0033 (8)	0.0069 (9)	−0.0077 (9)
C6	0.0475 (10)	0.0590 (11)	0.0575 (11)	−0.0019 (8)	0.0142 (8)	−0.0030 (9)
C7	0.0589 (12)	0.0756 (13)	0.0514 (10)	0.0145 (10)	0.0152 (9)	0.0050 (9)
C8	0.0675 (14)	0.0900 (17)	0.0931 (17)	0.0315 (13)	−0.0065 (12)	0.0054 (13)
C9	0.0573 (11)	0.0587 (11)	0.0490 (10)	0.0026 (9)	0.0164 (8)	0.0015 (8)
C10	0.0536 (11)	0.0596 (11)	0.0433 (9)	0.0034 (9)	0.0117 (8)	−0.0026 (8)
C11	0.0617 (12)	0.0680 (12)	0.0466 (10)	0.0098 (9)	0.0146 (9)	0.0029 (9)
C12	0.0527 (10)	0.0548 (10)	0.0465 (10)	0.0003 (8)	0.0061 (8)	−0.0044 (8)
C13	0.0606 (12)	0.0760 (13)	0.0525 (11)	0.0089 (10)	0.0131 (9)	0.0037 (9)
C14	0.0541 (12)	0.0737 (13)	0.0666 (12)	0.0029 (10)	0.0104 (9)	−0.0047 (10)
C15	0.0549 (11)	0.0563 (10)	0.0484 (10)	−0.0049 (8)	0.0006 (8)	−0.0058 (8)
C16	0.0811 (16)	0.0956 (16)	0.0524 (12)	0.0066 (13)	0.0046 (11)	0.0097 (11)
C17	0.0693 (16)	0.134 (2)	0.0976 (19)	0.0252 (16)	0.0270 (14)	0.0061 (17)

### Geometric parameters (Å, °)

**Table:**

S1—C14	1.716 (2)	C2—H2	0.9300
S1—C15	1.7063 (19)	C5—H5	0.9300
O1—C3	1.369 (2)	C6—H6	0.9300
O1—C7	1.417 (2)	C7—H7A	0.9600
O2—C4	1.372 (2)	C7—H7B	0.9600
O2—C8	1.425 (3)	C7—H7C	0.9600
O3—C11	1.220 (2)	C8—H8A	0.9600
C1—C2	1.407 (2)	C8—H8B	0.9600
C1—C6	1.382 (2)	C8—H8C	0.9600
C1—C9	1.459 (2)	C9—H9	0.9300
C2—C3	1.376 (2)	C10—H10	0.9300
C3—C4	1.405 (2)	C13—H13	0.9300
C4—C5	1.372 (3)	C16—H16A	0.9600
C5—C6	1.389 (3)	C16—H16B	0.9600
C9—C10	1.319 (3)	C16—H16C	0.9600
C10—C11	1.480 (2)	C16—H16D	0.9600
C11—C12	1.472 (3)	C16—H16E	0.9600
C12—C13	1.427 (3)	C16—H16F	0.9600
C12—C15	1.372 (2)	C17—H17A	0.9600
C13—C14	1.344 (3)	C17—H17B	0.9600
C14—C17	1.503 (3)	C17—H17C	0.9600
C15—C16	1.503 (2)		
			
S1···C1^i^	3.5635 (17)	H2···C7	2.5400
S1···C11^ii^	3.6293 (18)	H2···C10	2.7900
S1···C12^ii^	3.6734 (18)	H2···H7A	2.3600
S1···C7^iii^	3.624 (2)	H2···H7B	2.3000
O1···O2	2.5588 (19)	H2···H10	2.2800
O1···C2^iv^	3.335 (2)	H2···O1^vii^	2.8100
O1···C10^iv^	3.356 (2)	H5···C8	2.5400
O2···O1	2.5588 (19)	H5···H8A	2.3500
O3···C6^v^	3.175 (2)	H5···H8B	2.3200
O3···C16	2.865 (3)	H5···H16E^v^	2.5900
O1···H16C^vi^	2.7000	H6···H9	2.3500
O1···H10^iv^	2.7700	H6···O3^v^	2.4100
O1···H2^iv^	2.8100	H7A···C2	2.7600
O2···H13^iv^	2.6800	H7A···H2	2.3600
O2···H7B^iv^	2.8800	H7A···H16A^i^	2.5500
O3···H9	2.4300	H7A···H16D^i^	2.4100
O3···H16B	2.6700	H7B···C2	2.7700
O3···H16E	2.1800	H7B···H2	2.3000
O3···H16A	2.6600	H7B···O2^vii^	2.8800
O3···H6^v^	2.4100	H7B···C3^vii^	3.1000
C1···S1^i^	3.5635 (17)	H7B···C4^vii^	2.8100
C2···O1^vii^	3.335 (2)	H8A···C5	2.8000
C5···C8^viii^	3.475 (3)	H8A···H5	2.3500
C6···O3^v^	3.175 (2)	H8B···C5	2.7400
C7···S1^vi^	3.624 (2)	H8B···H5	2.3200
C8···C5^ix^	3.475 (3)	H8C···C5^ix^	2.9300
C10···C12^i^	3.598 (2)	H8C···C6^ix^	3.0800
C10···O1^vii^	3.356 (2)	H9···O3	2.4300
C11···S1^ii^	3.6293 (18)	H9···H6	2.3500
C12···C10^i^	3.598 (2)	H10···C2	2.8000
C12···S1^ii^	3.6734 (18)	H10···C13	2.6800
C16···O3	2.865 (3)	H10···H2	2.2800
C2···H7B	2.7700	H10···H13	2.1700
C2···H10	2.8000	H10···O1^vii^	2.7700
C2···H7A	2.7600	H13···C10	2.7600
C3···H7B^iv^	3.1000	H13···H10	2.1700
C3···H16C^vi^	2.9600	H13···H17A	2.6000
C4···H7B^iv^	2.8100	H13···O2^vii^	2.6800
C5···H8C^viii^	2.9300	H13···C8^vii^	3.0800
C5···H8A	2.8000	H16A···O3	2.6600
C5···H8B	2.7400	H16A···H7A^i^	2.5500
C6···H8C^viii^	3.0800	H16B···O3	2.6700
C7···H2	2.5400	H16B···C13^ii^	3.0400
C8···H13^iv^	3.0800	H16B···C14^ii^	2.9800
C8···H5	2.5400	H16C···O1^iii^	2.7000
C10···H13	2.7600	H16C···C3^iii^	2.9600
C10···H2	2.7900	H16D···H7A^i^	2.4100
C11···H16E	2.7800	H16E···C11	2.7800
C13···H10	2.6800	H16E···O3	2.1800
C13···H16F^ii^	2.8600	H16E···H5^v^	2.5900
C13···H16B^ii^	3.0400	H16F···C13^ii^	2.8600
C14···H16B^ii^	2.9800	H17A···H13	2.6000
			
C14—S1—C15	93.32 (9)	O1—C7—H7A	109.00
C3—O1—C7	117.93 (14)	O1—C7—H7B	109.00
C4—O2—C8	117.60 (16)	O1—C7—H7C	109.00
C2—C1—C6	118.52 (15)	H7A—C7—H7B	109.00
C2—C1—C9	122.25 (15)	H7A—C7—H7C	109.00
C6—C1—C9	119.17 (15)	H7B—C7—H7C	109.00
C1—C2—C3	120.42 (15)	O2—C8—H8A	110.00
O1—C3—C2	125.00 (15)	O2—C8—H8B	109.00
O1—C3—C4	114.88 (15)	O2—C8—H8C	109.00
C2—C3—C4	120.12 (16)	H8A—C8—H8B	109.00
O2—C4—C3	114.92 (15)	H8A—C8—H8C	109.00
O2—C4—C5	125.49 (16)	H8B—C8—H8C	109.00
C3—C4—C5	119.59 (16)	C1—C9—H9	115.00
C4—C5—C6	120.17 (16)	C10—C9—H9	115.00
C1—C6—C5	121.18 (16)	C9—C10—H10	119.00
C1—C9—C10	129.21 (17)	C11—C10—H10	119.00
C9—C10—C11	121.36 (16)	C12—C13—H13	123.00
O3—C11—C10	120.27 (17)	C14—C13—H13	123.00
O3—C11—C12	121.02 (17)	C15—C16—H16A	109.00
C10—C11—C12	118.71 (15)	C15—C16—H16B	109.00
C11—C12—C13	125.28 (16)	C15—C16—H16C	109.00
C11—C12—C15	123.34 (16)	C15—C16—H16D	109.00
C13—C12—C15	111.37 (17)	C15—C16—H16E	109.00
C12—C13—C14	114.71 (18)	C15—C16—H16F	109.00
S1—C14—C13	109.79 (15)	H16A—C16—H16B	109.00
S1—C14—C17	120.94 (15)	H16A—C16—H16C	109.00
C13—C14—C17	129.27 (19)	H16B—C16—H16C	109.00
S1—C15—C12	110.81 (14)	H16D—C16—H16E	109.00
S1—C15—C16	119.62 (14)	H16D—C16—H16F	109.00
C12—C15—C16	129.57 (17)	H16E—C16—H16F	109.00
C1—C2—H2	120.00	C14—C17—H17A	109.00
C3—C2—H2	120.00	C14—C17—H17B	109.00
C4—C5—H5	120.00	C14—C17—H17C	109.00
C6—C5—H5	120.00	H17A—C17—H17B	109.00
C1—C6—H6	119.00	H17A—C17—H17C	109.00
C5—C6—H6	119.00	H17B—C17—H17C	109.00
			
C15—S1—C14—C13	0.60 (19)	C2—C3—C4—C5	0.3 (3)
C15—S1—C14—C17	−179.89 (19)	O2—C4—C5—C6	178.31 (16)
C14—S1—C15—C12	−0.23 (15)	C3—C4—C5—C6	−0.6 (3)
C14—S1—C15—C16	179.43 (17)	C4—C5—C6—C1	0.1 (3)
C7—O1—C3—C2	6.1 (2)	C1—C9—C10—C11	−176.81 (16)
C7—O1—C3—C4	−172.97 (16)	C9—C10—C11—O3	−1.6 (3)
C8—O2—C4—C3	176.26 (17)	C9—C10—C11—C12	177.63 (16)
C8—O2—C4—C5	−2.7 (3)	O3—C11—C12—C13	175.23 (19)
C6—C1—C2—C3	−0.9 (2)	O3—C11—C12—C15	−4.6 (3)
C9—C1—C2—C3	176.38 (16)	C10—C11—C12—C13	−4.0 (3)
C2—C1—C6—C5	0.7 (2)	C10—C11—C12—C15	176.11 (16)
C9—C1—C6—C5	−176.74 (16)	C11—C12—C13—C14	−179.21 (19)
C2—C1—C9—C10	3.9 (3)	C15—C12—C13—C14	0.7 (3)
C6—C1—C9—C10	−178.80 (18)	C11—C12—C15—S1	179.70 (14)
C1—C2—C3—O1	−178.57 (15)	C11—C12—C15—C16	0.1 (3)
C1—C2—C3—C4	0.5 (2)	C13—C12—C15—S1	−0.2 (2)
O1—C3—C4—O2	0.4 (2)	C13—C12—C15—C16	−179.81 (19)
O1—C3—C4—C5	179.43 (15)	C12—C13—C14—S1	−0.8 (3)
C2—C3—C4—O2	−178.71 (15)	C12—C13—C14—C17	179.7 (2)

Symmetry codes: (i) −*x*, −*y*, −*z*; (ii) −*x*, −*y*+1, −*z*; (iii) *x*−1/2, −*y*+1/2, *z*−1/2; (iv) −*x*+1/2, *y*−1/2, −*z*+1/2; (v) −*x*+1, −*y*, −*z*; (vi) *x*+1/2, −*y*+1/2, *z*+1/2; (vii) −*x*+1/2, *y*+1/2, −*z*+1/2; (viii) −*x*+3/2, *y*+1/2, −*z*+1/2; (ix) −*x*+3/2, *y*−1/2, −*z*+1/2.

### Hydrogen-bond geometry (Å, °)

**Table:**

*D*—H···*A*	*D*—H	H···*A*	*D*···*A*	*D*—H···*A*
C6—H6···O3^v^	0.93	2.41	3.175 (2)	139

Symmetry codes: (v) −*x*+1, −*y*, −*z*.

## References

[bb1] Bandgar, B. P., Patil, S. A., Korbad, B. L., Biradar, S. C., Nile, S. N. & Khobragade, C. N. (2010). *Eur. J. Med. Chem.* **45**, 3223–3227. 10.1016/j.ejmech.2010.03.045 20430485

[bb2] Bernstein, J., Davis, R. E., Shimoni, L. & Chang, N.-L. (1995). *Angew. Chem. Int. Ed. Engl.* **34**, 1555–1573.

[bb3] Bruker (2005). *SADABS* Bruker AXS Inc., Madison, Wisconsin, USA.

[bb4] Bruker (2009). *APEX2* and *SAINT* Bruker AXS Inc., Madison, Wisconsin, USA.

[bb5] Deng, J., Sanchez, T., Al-Mawsawi, L. Q., Dayam, R., Yunes, R. A., Garofalo, A., Bolger, M. B. & Neamati, N. (2007). *Bioorg. Med. Chem.* **15**, 4985–5002. 10.1016/j.bmc.2007.04.041 17502148

[bb6] Farrugia, L. J. (1997). *J. Appl. Cryst.* **30**, 565.

[bb7] Farrugia, L. J. (1999). *J. Appl. Cryst.* **32**, 837–838.

[bb8] Liu, M., Wilairat, P., Croft, S. L., Tan, A. L. C. & Go, M. (2003). *Bioorg. Med. Chem.* **11**, 2729–2738. 10.1016/s0968-0896(03)00233-5 12788347

[bb9] Sheldrick, G. M. (2008). *Acta Cryst.* A**64**, 112–122. 10.1107/S0108767307043930 18156677

[bb10] Spek, A. L. (2009). *Acta Cryst.* D**65**, 148–155. 10.1107/S090744490804362X PMC2631630 19171970

[bb11] Verma, A. K., Koul, S., Pannu, A. P. S. & Razdan, T. K. (2007). *Tetrahedron*, **63**, 8715–8722.

